# Phosphinoborine Compounds: Mass Spectra and Pyrolysis*

**DOI:** 10.6028/jres.063A.004

**Published:** 1959-08-01

**Authors:** Leo A. Wall, Sidney Straus, Roland E. Florin, Fred L. Mohler, Paul Bradt

## Abstract

The mass spectra of tetramethylphosphinoborine trimer, [P(CH_3_)_2_B(CH_3_)_2_]_3_ (I) and a a compound, P_5_(CH_3_)_9_B_5_H_9_, (II) prepared from dimethylphosphinoborine were observed, and the compounds were pyrolyzed at 300 to 500° C. Most peaks in the spectrum of (I) came from the P—B, B—C, and P—C cleavages. The mass spectrum of (II) was much more complicated with evidence for methyl group redistribution.

The pyrolysis of both compounds indicates a very complicated mechanism with many unidentifiable compounds. Trends in the formation of volatile products indicate that both compounds are completely decomposed in 4 hr at 450° C. Compound (I) produces trimethylboron, which disappears rapidly above 400° C. Neither (I) nor (II) formed ethane or elemental phosphorus.

## 1. Introduction

The results of mass spectrometric and pyrolytic studies on the trimer of dimethylphosphinoborine have previously been reported.^1^ In this present article results on two related compounds^2^ are presented. They are a trimer of tetramethylphosphinoborine (I) and a pentameric form of dimethylphosphinoborine (II),^3^ which has lost a molecule of methane.

**Figure:**
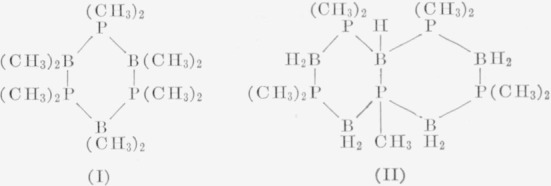


Their structures are believed to be composed of cyclic six-membered rings as shown.

## 2. Mass Spectra

The phosphinoborine compounds were not volatile enough to be run in the gas-analysis instrument, and the mass spectra were obtained in a 60° “Nier” instrument with the sample evaporated directly into the ionization chamber. This technique has been described in other papers.^4^

The sample was held in a small tube furnace. The trimer (I) attained a temperature of about 54° C due to heat from the mass spectrometer filament and evaporated copiously without external heating. The ion current for the most abundant ion at mass 143 in terms of the most sensitive scale was 42,800 scale divisions at 50 volts ionizing voltage and 35,000 scale divisions at 20 volts. The sample became exhausted before measurements with 15 volts ionizing voltage were completed.

The compound (II), at 54° C and 50 volts ionizing voltage, gave an ion current of 123 divisions at mass 340; with 20 volts and a slightly higher temperature the current was 184 divisions; and with 15 volts and slightly increased temperature the current was 1,176 divisions.

Table 1 gives results for the trimer (I). The mass spectrum is complicated by the isotopic structure of boron atoms as well as by the C^13^ isotope of carbon. The monoisotopic spectrum has been computed,^5^ and the mass scale pertains to molecules containing B^11^, C^12^, and H. In the computation it is assumed that, the abundance ratio of boron isotopes is B^10^, 0.20; B^11^, 0.80. This is a relatively simple spectrum for a molecule containing 54 atoms. There is no appreciable dissociation of H atoms in the process of ionization. Because of this, one can see immediately from the isotopic structure the number of B atoms in each fragment ion. A number of small peaks of 1 or 2 percent of the maximum peak are not accounted for, but all the larger peaks are explained by breaking of P—B, C—B, and C—P bonds.

The monoisotopic spectrum of compound (II) is very complicated. In the ionization process, hydrogen atoms are readily removed and this conceals the isotope structure of the resulting ions. One can identify the more abundant light ions up to mass 89 unambiguously, and by trial one can find molecular formulas for the heavy ions that fit the data without negative residuals. In the intermediate mass range from 90 to 260 the molecular constitution of the ions has not been established, and this part of the mass spectrum is omitted from table 2. This spectrum is in marked contrast to that in table 1. The molecular ion is fairly abundant, and the breaking of B—H, B—P, and P—C bonds occurs in the ionization process. Ions heavier than 261 occur in nearly the same relative intensity at 20 volts as at 50 volts, indicating that the appearance potentials for most of the ions fall in a narrow range of voltage and are far below 20 volts.

There is some resemblance between the mass spectra of compound (II) and the trimer (see footnote 1) of dimethylphosphinoborine although relative intensities are quite different. The trimer (I) studied in this work gives a very different type of spectrum. The molecule ion is much less abundant in this case and there is no tendency to lose H atoms in the ionization process. Evidently loss of H atoms comes predominantly from B–H bonds, and the chemical formulas of the ions in table 2 support this. Compound (II) can lose 9 H atoms and no more.

## 3. Pyrolysis

Since the total quantity of each compound available for study was only of the order of 0.1 g, and since it was desired to obtain some quantitative data on the thermal stability of these compounds, a technique was tried in which an inert rare gas, argon, was used as an internal standard. Into each of a series of 8-ml tubes was pipetted a given amount of an accurately known benzene solution of the compound to be studied. Before sealing the tubes on a vacuum manifold the benzene solvent was evaporated. Each of the sample tubes thus contained 3.00 mg of material.

Generally eight of these tubes plus a gas-sample tube could be attached to a vacuum line manifold and evacuated overnight to better than 10^−4^ mm of Hg by means of an oil pump, an Hg diffusion pump, and a liquid nitrogen trap. For purification of the argon a silica-gel trap, also in this vacuum line, was first heated to about 280 to 290° C to eliminate any impurities that might be absorbed in the gel. The following day the heater was removed from the silica-gel trap and soon afterwards crushed dry ice was placed in a Dewar around the silica gel.

An Hg manometer was used to read known volumes of argon gas introduced in the system. The argon gas was slowly passed through the cooled silica gel, and a predetermined amount of the gas was permitted to enter the ampoules containing the samples. The number of moles of argon introduced was approximately 3 times the number of moles of material to undergo pyrolysis. At this point the 8 ampoules and the gas-sample tube were sealed off by means of an oxygen flame at the 3-mm glass tube extensions from the manifold.

Prior to pyrolysis the gas-sample tube was first analyzed in the mass spectrometer determining the purity of the argon gas. Then the weighed amounts of sample and known volumes of gas in the glass ampoules were pyrolyzed in a large copper furnace at temperatures maintained to within about ±1° C. Pyrolysis experiments were made at various temperatures for different periods of time, generally 2, 4, 8, and 24 hr. Mass spectra determinations of the volatile products were then made. Since each tube contained a known amount of argon, these mass spectrometric analyses could be used to calculate the number of moles or grams of each of the volatile products.

## 4. Results and Discussion

**Figure 1:**
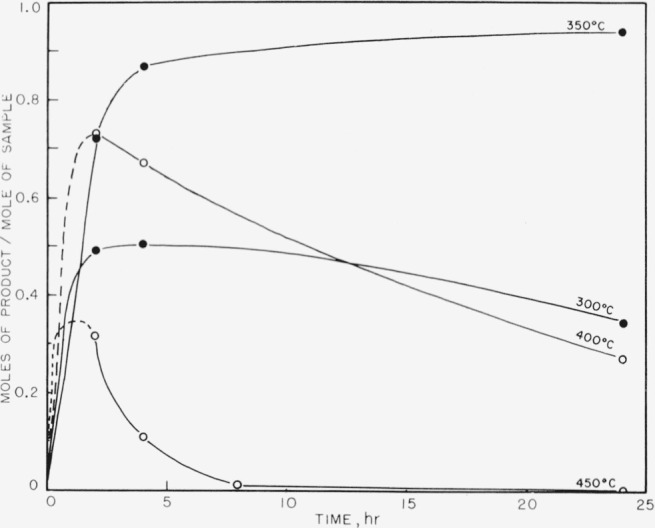
Boron trimethyl production from the thermal decomposition of *P*_3_*B*_3_(*CH*_3_)_12_.

**Figure 2:**
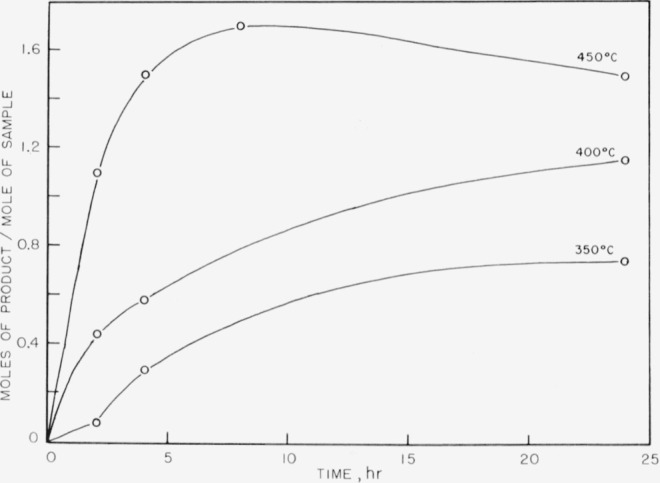
Methane production from the thermal decomposition of *P*_3_*B*_3_(*CH*_3_)_12_.

**Figure 3:**
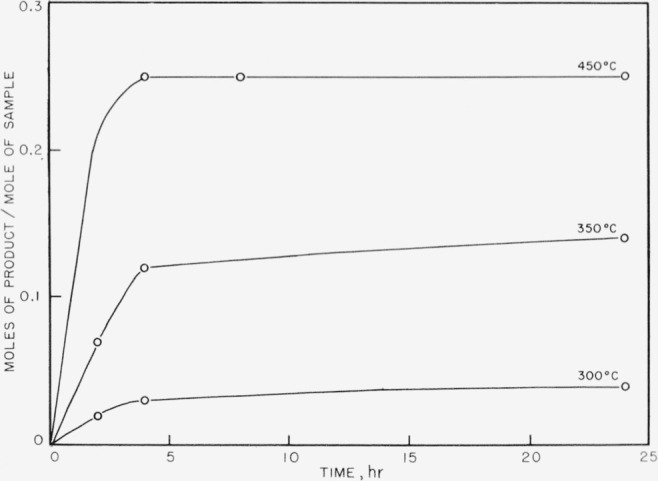
Hydrogen production from the thermal decomposition of *P*_3_*B*_3_(*CH*_3_)_12_.

**Figure 4:**
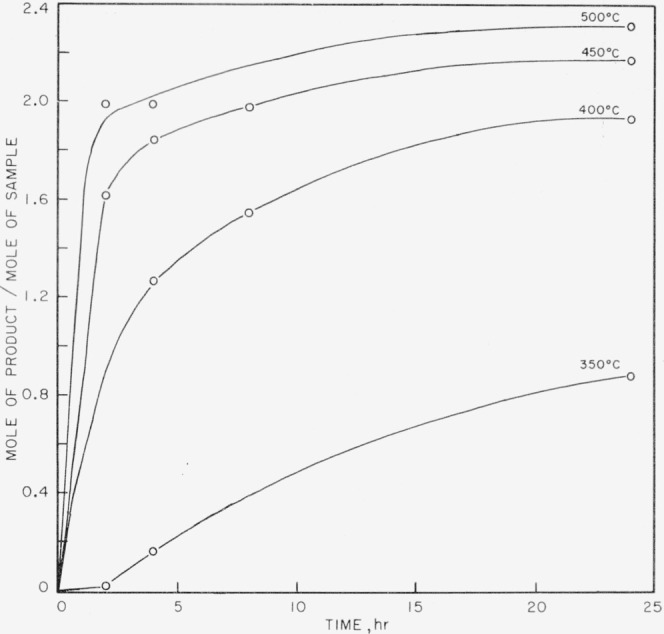
Methane production from the thermal decomposition of *P*_5_ (*CH*_3_)_9_*B*_5_*H*_9_.

**Figure 5:**
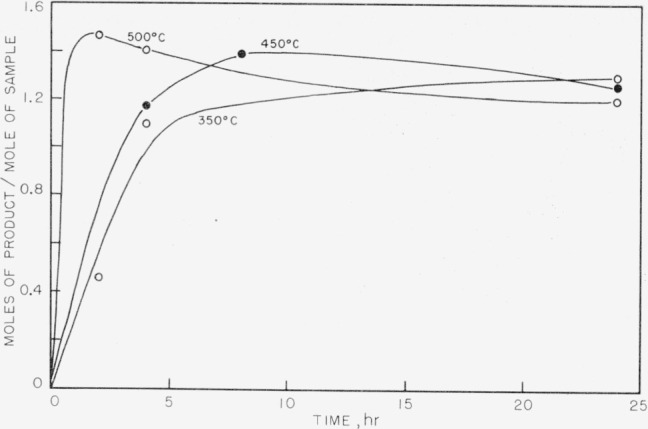
Hydrogen production from the thermal decomposition of *P*_5_(*CH*_3_)_9_*B*_5_*H*_9_.

The products obtained on pyrolysis were very complex mixtures, and only hydrogen, methane, and trimethyl boron were identified by mass spectrometer analysis. The trimethyl boron was obtained only in the breakdown of the completely methylated P–B compound. The results are shown in figures 1 to 5. In all cases the quantitative yields of volatiles computed were based on the known pressure of argon gas present initially and the determined mole percent of argon as shown by mass spectrometry for each set of temperatures and times of pyrolysis. These samples also contained boron compounds with peaks up to mass 126 that could not be readily identified. These boron compounds accounted for approximately 5 percent of the total volatiles in the sample.

In figure 1 trimethyl boron is shown as one of the initial products from compound (I) and is apparently produced readily at 300° and at 350° C. At higher temperatures the amount of trimethyl boron decreases rapidly, indicating further breakdown. On the other hand, methane and hydrogen (see figures 2 and 3) achieve maximum yields at 450° C. No elemental phosphorus was seen among the decomposition products, in contrast to the previous work on the dimethylphosphinoborine trimer (see footnote 1).

In figures 4 and 5 the methane and hydrogen from (II) are shown. Trimethyl boron was not produced. It is fairly clear from the structure that any production of trimethyl boron is unlikely because of the great amount of rearrangement which would be required. Little or no ethane was found from the decomposition of either of the two phosphinoborine compounds studied. The second substance produced greater amounts of methane and hydrogen than the all-methylated compound, which seems reasonable in view of the difference in structure and composition.

If the maximum possible number of trimethyl boron molecules come from a decomposed starting molecule, then at least 31 percent of the original material has decomposed after 24 hr at 350° C. The trends in the product formation however suggest that after 24 hr at 350° C little of either of the original phosphinoborine compounds remains. Subsequent heating presumably causes further decomposition of the products. It appears that roughly one molecule of trimethyl boron is produced from each molecule of starting material. The production of the trimethyl boron means that the ring structure is ruptured.

The second compound was pyrolyzed at temperatures as high as 500° C with no indication of elemental phosphorus being present in the decomposition products. Previous work (see footnote 1) on related compounds showed that phosphorus was produced at 500° C pyrolysis temperatures. The yield of methane and hydrogen, however, was high enough that a rather large degree of decomposition must have occurred. Although no identifiable compound was observed containing boron or phosphorus, some boron compounds were present in the volatile products, indicating again a rupture in the ring.

The results indicate a very complicated mechanism of decomposition, with a great number of products, most of which are not very volatile and some of which are not identifiable because of the lack of mass spectrometric data on organo boron-phosphorus compounds. It seems apparent from the trends in the yields of the identified products that the original material in the case of both substances is exhausted in a matter of about 4 hr at 450° C. It is impossible at this time to distinguish any difference between the thermal stabilities of the two compounds.

Using the initial slopes in the experiments, very rough estimates of the activation energies for methane and hydrogen production were made. For compound (I) these were found, respectively, to be 29 and 12 kcal/mole, and for compound (II), 4 and 7 kcal/mole.

It is worth noting that ethane was essentially absent among the pyrolysis products. This suggests that if methyl radicals were intermediate species for the formation of methane, they reacted very rapidly, as one might expect, to abstract hydrogen atoms from the compounds. On the other hand, molecular rearrangement processes not involving free-radical intermediates may account for the results.

## Figures and Tables

**Table 1:** Monoisotopic spectrum of $B_{3}^{11}{\left({\mathrm{CH}}_{3}\right)}_{6}P_{3}{\left({\mathrm{CH}}_{3}\right)}_{6}$

m/e	Ion	50 volts	20 volts
			
41	B(CH_3_)_2_	30.5	1.61
46	P(CH_3_)	.96	.52
61	P(CH_3_)_2_	.55	.17
62	PH(CII_3_)_2_	.85	.57
63	PH_2_(CH_3_)_2_	.90	.05
87	BP(CH_3_)_3_	1.95	.36
102	BP(CH_3_)_4_	2.7	.47
103	BPH(CH_3_)_4_	10.9	2.02
113	B_2_P(CH_3_)_4_	1.06	…………
129	B_2_PH(CH_3_)_5_	5.3	2.44
143	B_2_P(CH_3_)_6_	100	100
159	B_2_P_2_(CH_3_)_5_	1.07	.27
163	BP_2_(CH_3_)_6_	.58	.22
189	B_2_P_2_(CH_3_)_7_	16.4	21.7
245	B_3_P_2_(CH_3_)_10_	27.6	59.3
291	B_3_P_3_(CH_3_)_11_	8.3	17.6
306	B_3_P_3_(CH_3_)_12_	.76	.99

**Table 2:** Monoisotopic mass spectrum of *B_5_H_9_P_5_(CH_3_)_9_*

m/e	Ion	50 volts	20 volts	15 volts
				
41	B(CH_3_)_2_	21.7	0.05	0.05
61	P(CH_3_)_2_	4.6	.45	.25
75	BH_3_P(CH_3_)_2_	17.5	…………..	…………..
89	BHP_2_(CH_3_)	14	…………..	…………..
(a)	(a)	(a)	(a)	(a)
261	B_4_H_3_P_4_(CH_3_)_6_	4.9	4.4	…………..
262	B_4_H_4_P_4_(CH_3_)_6_	5.1	5.4	1.95
263	B_4_H_5_P_4_(CH_3_)_6_	12.4	14.4	4.15
264	B_4_H_6_P_4_(CH_3_)_6_	1.45	1.21	0
265	B_4_H_7_P_4_(CH_3_)_6_	3.0	3.2	1.05
273	B_4_P_4_(CH_3_)_7_	5.15	8.0	1.10
274	B_4_HP_4_(CH_3_)_7_	1.90	1.53	0.55
275	B_4_H_2_P_4_(CH_3_)_7_	9.25	9.10	3.0
276	B_4_H_3_P_4_(CH_3_)_7_	4.75	4.82	1.65
277	B_4_H_4_P_4_(CH_3_)_7_	14.3	14.9	5.75
278	B_4_H_5_P_4_(CH_3_)_7_	5.90	5.85	3.6
279	B_4_H_6_P_4_(CH_3_)_7_	7.60	7.90	4.25
287	B_5_H_3_P_4_(CH_3_)_7_	6.25	5.90	2.0
288	B_5_H_4_P_4_(CH_3_)_7_	0.95	0.85	0
289	B_5_H_5_P_4_(CH_3_)_7_	19.9	20.0	11.9
290	B_5_H_6_P_4_(CH_3_)_7_	3.9	4.0	2.15
291	B_5_H_7_P_4_(CH_3_)_7_	9.2	11.0	7.5
292	B_5_H_8_P_4_(CH_3_)_7_	4.1	3.56	3.9
293	B_5_H_9_P_4_(CH_3_)_7_	5.5	7.1	5.25
322	B_4_H_3_P_5_(CH_3_)_8_	1.76	1.35	…………..
323	B_4_H_4_P_5_(CH_3_)_8_	2.54	2.07	2.3
324	B_4_H_5_P_5_(CH_3_)_8_	36.6	35.1	39.0
325	B_4_H_6_P_5_(CH_3_)_8_	19.0	16.2	18.0
326	B_4_H_7_P_5_(CH_3_)_8_	7.7	8.5	9.4
327	B_4_H_8_P_5_(CH_3_)_8_	2.62	1.67	1.85
334	B_4_P_5_(CH_3_)_9_	1.08	1.13	0.90
335	B_4_HP_5_(CH_3_)_9_	4.90	4.50	2.45
336	B_4_H_2_P_5_(CH_3_)_9_	3.30	2.43	2.20
337	B_4_H_3_P_5_(CH_3_)_9_	3.84	3.96	4.40
338	B_4_H_4_P_5_(CH_3_)_9_	19.4	19.4	19.5
340	B_4_H_6_P_5_(CH_3_)_9_	100	100	100
347	B_5_H_2_P_5_(CH_3_)_9_	2.72	2.34	1.70
348	B_5_H_3_P_5_(CH_3_)_9_	2.48	0.95	2.15
349	B_5_H_4_P_5_(CH_3_)_9_	4.15	2.48	1.50
350	B_5_H_5_P_5_(CH_3_)_9_	17.9	16.6	20.5
351	B_5_H_6_P_5_(CH_3_)_9_	4.1	1.80	3.35
352	B_5_H_7_P_5_(CH_3_)_9_	87.	84.1	105
353	B_5_H_8_P_5_(CH_3_)_9_	3.84	2.48	3.05
354	B_5_H_9_P_5_(CH_3_)_9_	26.4	18.0	13.9

a Mass range 90 to 260 omitted. Ions have not been identified.

## Footnotes

1 R. E. Florin, L. A. Wall, F. L. Mohler, and E. Quinn. J. Am. Chem. Soc. **76**, 3344 (1954).

2 The authors wish to thank Dr. R. I. Wagner of the American Potash Co. for kindly supplying the samples used in this work.

3 A. B. Burg and R. I. Wagner, J. Am. Chem. Soc. **75**, 3872 (1953).

4 Paul Bradt and Fred L. Mohler, J. Research NBS **55**, 323 (1955).

5 V. H. Dibeler, F. L., Mohler, L. Williamson, and R. M. Reese, J. Research NBS **43**, 97 (1949).

